# Taxonomic and phenotypic characterization of a novel *Providencia* species: *Providencia lanzhouensis* sp. nov.

**DOI:** 10.1128/spectrum.00549-25

**Published:** 2025-07-09

**Authors:** Yanghui Xiang, Xu Dong, Lan Ma, Dan Cao, Yi Li, Xiuzhi Jiang, Pusheng Xu, Xin Yuan, Kefan Bi, Yiru Zhang, Yuxin Han, Ying Zhang

**Affiliations:** 1 State Key Laboratory for Diagnosis and Treatment of Infectious Diseases, National Clinical Research Center for Infectious Diseases, China-Singapore Belt and Road Joint Laboratory on Infection Research and Drug Development, National Medical Center for Infectious Diseases, Collaborative Innovation Center for Diagnosis and Treatment of Infectious Diseases, The First Affiliated Hospital, Zhejiang University School of Medicine, Zhejiang University School of Medicine 26441 , Hangzhou, Zhejiang, China; 2 Department of Laboratory Medicine, The Second Hospital & Clinical Medical School, Lanzhou University 12426, Lanzhou, Gansu, China; 3 Jinan Microecological Biomedicine Shandong Laboratory 661980 , Jinan, China; Institute of Microbiology, Chinese Academy of Sciences, Beijing, China

**Keywords:** *Providencia*, novel species, taxonomy, ANI, antimicrobial resistance

## Abstract

The genus *Providencia* includes species of ecological and clinical significance, with several members acting as opportunistic pathogens in hospital-acquired infections, particularly urinary tract infections (UTIs). However, overlapping phenotypic traits and genetic similarities among species pose challenges for accurate identification. In this study, we isolated strain PAZ2 from a urine sample of a hospitalized patient in Lanzhou, Gansu Province, China. While initial mass spectrometry analysis identified the strain as *Providencia alcalifaciens*, comprehensive genomic analysis revealed distinct characteristics suggesting a novel species. Comparative genomic analyses showed average nucleotide identity (ANI) values ranging from 79.92% to 94.73% and digital DNA-DNA hybridization (dDDH) values between 21.2% and 57.6% when compared with known *Providencia* species. The strain exhibited resistance to multiple antibiotics, including ampicillin, tetracycline, tigecycline, polymyxin B, colistin, trimethoprim-sulfamethoxazole, and ciprofloxacin. Biochemical characterization revealed distinct properties that differentiated PAZ2 from previously described species within the genus. Based on genomic and phenotypic evidence, we propose strain PAZ2 as the type strain of a novel species, *Providencia lanzhouensis* sp. nov. Geographic distribution analysis indicated *P. lanzhouensis* presence in five countries, with predominance in China. Notably, comparative genomic analysis revealed that *P. lanzhouensis* PAZ2 harbors an expanded repertoire of antimicrobial resistance genes compared to other *Providencia* strains with high ANI values. This discovery expands our understanding of *Providencia* taxonomy and highlights its clinical significance. Further studies investigating its pathogenicity mechanisms, antimicrobial resistance profiles, and epidemiological patterns will be crucial for developing effective treatment strategies.

**IMPORTANCE:**

Accurate identification of bacterial pathogens is crucial for effective clinical treatment, yet current diagnostic methods sometimes fall short when encountering novel species. This study describes *Providencia lanzhouensis*, a new bacterial species isolated from a clinical setting in China, which was initially misidentified by conventional methods. Our discovery not only expands the known diversity of the *Providencia* genus but also reveals important insights about antimicrobial resistance in emerging pathogens. *P. lanzhouensis* carries multiple resistance genes, highlighting potential clinical challenges and the need for accurate identification in healthcare settings. This work demonstrates the importance of integrating multiple analytical approaches for bacterial identification and underscores the dynamic nature of bacterial evolution. These findings have significant implications for improving pathogen identification systems and developing effective treatment strategies in clinical practice.

## INTRODUCTION

The *Providencia* genus comprises facultatively anaerobic, Gram-negative bacilli, originally classified within the *Enterobacteriaceae* family but subsequently reassigned to the *Morganellaceae* family following reclassification within the order *Enterobacterales* (1). Species within the *Providencia* genus are widely distributed in diverse environmental settings, including water, soil, and various animal hosts (2). These organisms are recognized as opportunistic pathogens, implicated in a range of clinical infections such as diarrhea, foodborne illnesses, urinary tract infections (UTIs), and bloodstream infections (3).

The taxonomy of *Providencia* remains complex and controversial. According to the latest update from the List of Prokaryotic names with Standing in Nomenclature (LPSN) (https://lpsn.dsmz.de/genus/providencia), the genus currently includes 15 validly published species, such as *Providencia stuartii*, *P. manganoxydans*, *P. burhodogranariea*, *P. sneebia*, *P. rettgeri*, *P. huaxiensis*, *P. vermicola*, *P. alcalifaciens*, *P. heimbachae*, *P. rustigianii*, *P. huashanensis*, *P. thailandensis*, *P. xianensis*, *P. zhijiangensis*, and *P. hangzhouensis*. Additionally, four species—*P. entomophila*, *P. wenzhouensis*, *P. zhejiangensis*, and *P. xihuensis*—have been proposed but are not yet validly published (4–19). Our previous genomic analyses have revealed that some of these species represent taxonomic synonyms. For instance, *P. stuartii* and *P. thailandensis*, as well as *P. xianensis* and *P. huashanensis*, have been shown to be synonymous species pairs based on comprehensive genome-based evidence (15, 19). Among the recognized species, *P. rettgeri*, *P. stuartii*, *P. alcalifaciens*, and *P. hangzhouensis* are particularly notable due to their clinical significance. *P. stuartii* is notably associated with UTIs and healthcare-associated infections, particularly among patients with prolonged indwelling urinary catheters, thus representing a significant pathogen in nosocomial settings (5). Another notable species, *P. rettgeri,* is also isolated from environmental sources like water and soil and has been linked to UTIs, diarrheal diseases, wound infections, and bacteremia (20). This species presents a heightened infection risk for immunocompromised individuals and is frequently associated with secondary infections in burn patients.

In the 19th century, classification of *Providencia* species relied primarily on physiological characteristics and biochemical reactions. However, advances in molecular biology have led to the adoption of increasingly precise techniques for species identification and differentiation, particularly where biochemical profiles may overlap among species. Among these, 16S rRNA gene sequencing due to its high conservation across bacterial taxa has been instrumental in accurately identifying *Providencia* species, especially when conventional biochemical methods prove insufficient. Despite its utility, 16S rRNA sequencing has limitations in resolving intraspecies diversity (21). Whole-genome sequencing (WGS), an advanced molecular tool, offers comprehensive insights into intraspecies variability and bacterial evolutionary patterns, establishing itself as the most robust method for accurate species identification.

In this study, we isolated a *Providencia* strain from a clinical sample collected at a tertiary hospital in Lanzhou. Initial identification using MALDI-TOF MS suggested the strain to be *P. alcalifaciens*. However, upon further analysis of distinct biochemical properties and whole-genome sequencing, we determined that this strain does not correspond to any known species within the *Providencia* genus. Therefore, we propose it as a novel species within the genus, tentatively named *Providencia lanzhouensis* sp. nov.

## MATERIALS AND METHODS

### Strains and antibiotic susceptibility testing

Strain PAZ2 was isolated from the urine sample of a patient at a Lanzhou hospital as part of a routine clinical microbiology practice. Primary species identification of the strain was conducted using MALDI-TOF MS (bioMérieux). The antimicrobial susceptibility of the isolate was evaluated using agar dilution method to establish the minimum inhibitory concentrations (MICs), following protocols outlined in CLSI standards (22). The interpretation of susceptibility breakpoints adhered to CLSI recommendations, with the exception of tigecycline assessment, which was conducted in accordance with EUCAST (European Committee on Antimicrobial Susceptibility Testing) parameters.

### Whole-genome sequencing and analysis

Bacterial DNA extraction was performed on this strain utilizing commercial DNA isolation reagents (QIAamp DNA Mini Kit manufactured by Qiagen). The genome sequencing strategy employed multiple platforms to ensure comprehensive coverage: short-read sequencing was conducted on an Illumina HiSeq 2500 platform to generate 150 bp paired-end reads, while long-read data were obtained using PacBio Sequel II technology. The integration of these complementary data sets was accomplished through the Unicycler pipeline (23) to construct complete genome assemblies. Taxonomic characterization involved comparative genomic analyses. Genome similarity metrics were calculated through two approaches: Average Nucleotide Identity (ANI) was computed using fastANI v1.32 (24), while digital DNA-DNA hybridization (dDDH) values were determined via the genome-to-genome distance calculator, implementing formula 2 (25). Species boundaries were established based on accepted thresholds (ANI ≥ 96% and dDDH ≥70.0%) (26).

The genomic analysis pipeline included screening for antimicrobial resistance determinants using multiple specialized tools and databases: ABRicate (https://github.com/tseemann/abricate), AMRFinderPlus (27). Genetic element identification and functional annotation were performed using the Bakta v1.9. 4 (28).

### Phylogenetic analysis

Amplification of the 16S rRNA gene was performed by PCR using universal primers 27F (5′-AGAGTTTGATCCTGGCTCAG-3′) and 1492R (5′-GGTTACCTTGTTACGACTT-3′) (29). Reference sequences for comparative analysis were retrieved from EzBioCloud (30) and type strain genome repositories. Sequence organization and refinement involved MAFFT v7.505 (31) for initial alignment, followed by optimization with trimal v1.4 (32). To enhance phylogenetic resolution, a multi-gene approach was implemented by analyzing five conserved housekeeping genes: *fusA* (encoding translational elongation factor EF-G), *gyrB* (DNA gyrase subunit B), *ileS* (isoleucyl-tRNA synthetase), *lepA* (translation elongation factor EF-4), and *leuS* (leucyl-tRNA synthetase). These sequences were extracted and combined from the study strains and relevant type strains and then processed through MAFFT and trimal pipelines. Genomic variation analysis was conducted through single-nucleotide polymorphism (SNP) detection using the Snippy v4.16 (https://github.com/tseemann/snippy). Evolutionary relationships were reconstructed using maximum likelihood methodology in FastTree (33), with phylogenetic tree visualization accomplished through ggtree (34). Panaroo v1.5.1 (35) with default parameters was used for pan-genome comparison.

### Phenotypic characterization

As described in our previous study (16), Gram staining and biochemical characterization were performed using the bioMérieux API 20E and API 50CH systems according to the manufacturer’s standardized protocols. Oxidase activity was assessed using bioMérieux’s oxidase test reagent. Cellular morphology was examined under an optical microscope following 16 h of incubation on LB agar at 37°C. Growth characteristics were evaluated on various media, including tryptic soy agar, Luria-Bertani agar, blood heart infusion agar, and Müller-Hinton agar. Optimal growth conditions were systematically assessed in tryptic soy broth, varying temperature (4–44°C), pH (4.0–11.0), and salinity (0–9%, wt/vol) within a controlled environment. Anaerobic growth potential was examined on BHI agar under anaerobic conditions for 48 h. Catalase activity was determined by adding 3% hydrogen peroxide (vol/vol) to fresh bacterial biomass from LB agar cultures followed by observing for the presence of effervescence.

### Global distribution of the novel species

To evaluate species delineation and genomic diversity, we retrieved all *Providencia* genomes available in the NCBI GenBank database (as of 31 December 2024). To determine the global distribution of the novel species, all genomes in the NCBI GenBank database (as of 31 December 2024) were screened via the fastANI.

## RESULTS

### Clinical information and *in vitro* drug susceptibility test

The PAZ2 strain was isolated on 21 March 2024 from a urine sample of a patient presenting with urinary tract infection (UTI) symptoms, including increased frequency and urgency of urination, as well as dysuria that had persisted for over one month. The patient had a known history of prostatic hyperplasia and a malignant tumor of the renal pelvis. Symptomatic and supportive treatment was administered during hospitalization. However, the patient declined surgical intervention and was subsequently discharged.

Strain PAZ2 was identified as *P. alcalifaciens* by MALDI-TOF MS, scoring 7.317 points. Following isolation and purification, the strain underwent comprehensive *in vitro* drug susceptibility testing. PAZ2 exhibited a narrow resistance profile, displaying resistance only to the antibiotics ampicillin, tetracycline, tigecycline, polymyxin B, colistin, trimethoprim-sulfamethoxazole, and ciprofloxacin. Furthermore, the strain demonstrated susceptibility to a range of additional antibiotics, including ceftazidime, cefepime, imipenem, meropenem, amikacin, and gentamicin (see Table 1).

**TABLE 1:** Antibiotic susceptibilities of the strain PAZ2

Antibiotics	PAZ2	
	MIC (mg/L)	Category^*a*^
Ampicillin	>128	R
Ceftazidime	<0.06	S
Cefepime	<0.06	S
Imipenem	0.05	S
Meropenem	<0.06	S
Amikacin	8	S
Gentamicin	2	S
Aztreonam	<0.06	S
Tetracycline	64	R
Tigecycline	8	R
Polymyxin B	4	R
Colistin	>128	R
Trimethoprim-sulfamethoxazole	>128	R
Ciprofloxacin	32	R

^*a*^ S, susceptible; R, resistant.

### Basic genomic features of strain PAZ2

**Fig 1:**
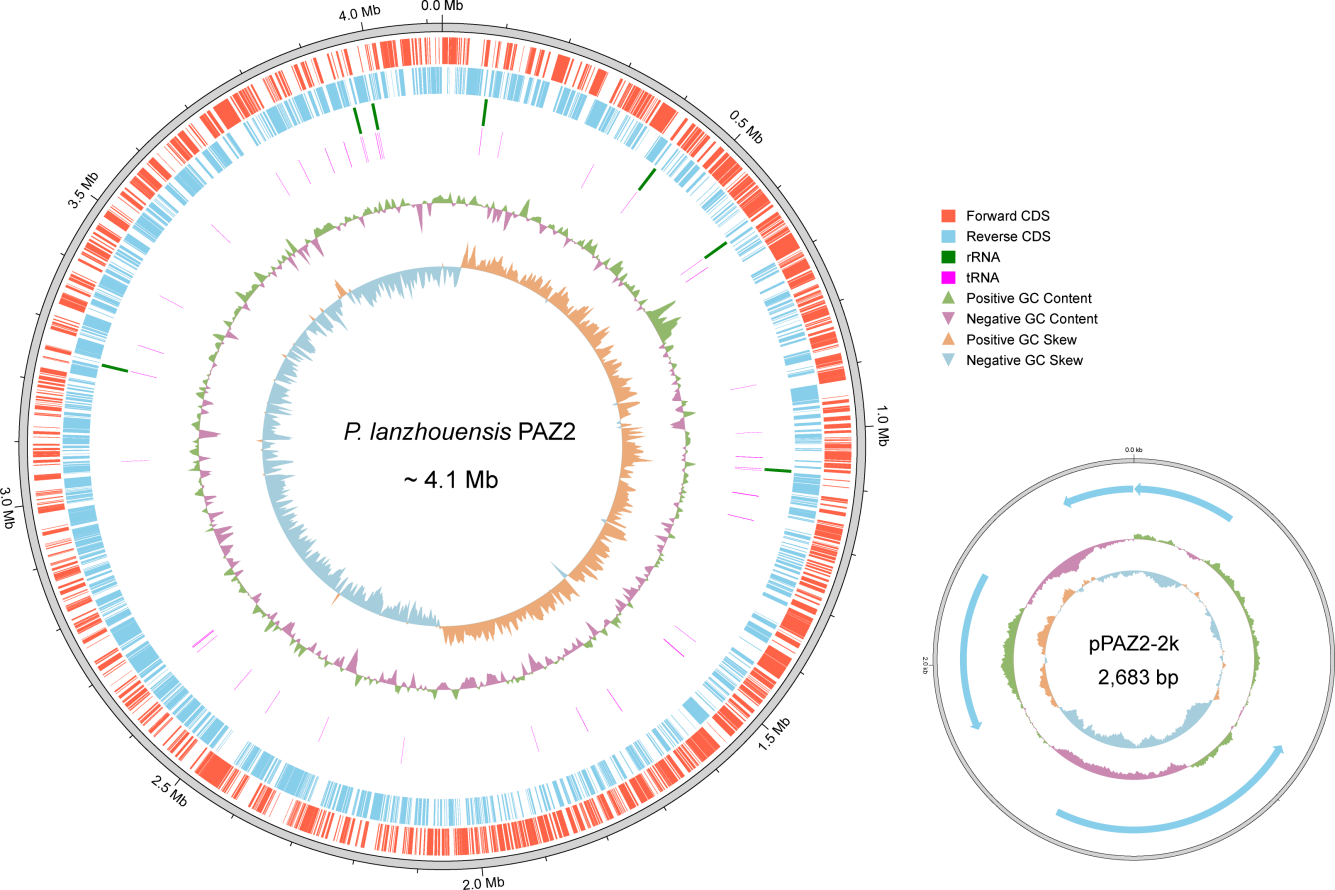
Structural diagrams of the chromosome and plasmid of PAZ2 identified in this study.

To characterize this strain and its representative species, we performed whole-genome sequencing analysis of the strain PAZ2. The complete genome consists of a 4.12 Mb chromosome and a 2,683 bp plasmid, with an overall G + C content of 42.8% (Fig. 1). Genome analysis revealed 12 antimicrobial resistance genes, with 11 chromosomal and 1 plasmid-borne gene. The chromosomal resistance genes encode resistance to multiple antibiotic classes: aminoglycosides (*sat2*, *aadA1*, *aph(6)-Id*, *aph(3'')-Ib*, *aph(3')-Ia*, *aadA2*), sulfonamides (*sul2*), macrolides (*ere(A*)), trimethoprim (*dfrA32*), phenicols (*floR*), and tetracyclines (*tetC*). The plasmid-encoded *qnrD1* gene confers resistance to quinolones. MOB-suite analysis identified the plasmid as a Col3M-like replicon, classified as non-mobilizable, suggesting limited potential for conjugative transfer. Further inspection revealed that all chromosomal resistance genes are associated with insertion sequences or integrons (Fig. S1), indicating acquisition through horizontal gene transfer. In contrast, the *qnrD1* gene on the plasmid lacks adjacent insertion sequences. This plasmid, a small non-conjugative element, shows 99.89% sequence identity and 97.69% coverage with the *qnrD1*-carrying plasmid pDIJ09-518a previously reported in *Providencia rettgeri* (36).

### Taxonomic analysis reveals PAZ2 as a novel species distinct from known species

**Fig 2:**
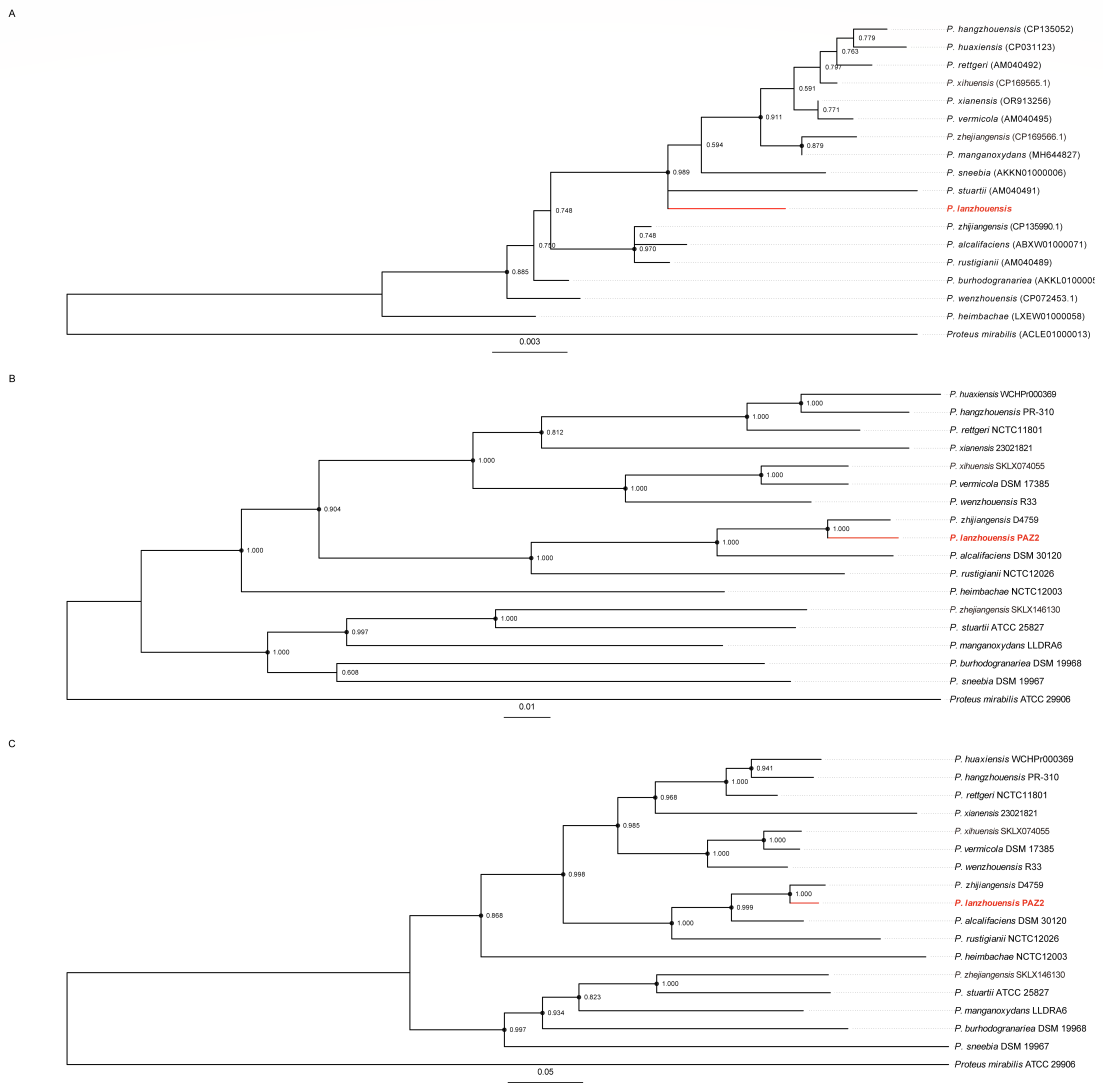
The evolutionary relationships within the *Providencia* genus were reconstructed through three distinct phylogenetic approaches. Panel (**A**) demonstrates the evolutionary framework inferred from 16S rRNA gene sequence analysis. Panel (**B**) represents phylogenetic associations derived from the concatenated sequences of five conserved housekeeping genes. Panel (**C**) illustrates the evolutionary patterns revealed through whole-genome SNP analysis. *Proteus mirabilis* ATCC 29906 serves as the designated outgroup species. The reliability of branching patterns was evaluated through 1,000 bootstrap replicates, with nodes displaying support values exceeding 50% being indicated. Notably, nodes with robust bootstrap support (>80%) are distinctly marked by solid black circles. Strains isolated and characterized in the present investigation are emphasized in red coloration.

To establish the taxonomic position of strain PAZ2, we conducted a comprehensive phylogenetic analysis using multiple molecular approaches. A BLASTn search of the 16S rRNA sequences of PAZ2 against the rRNA_typestrains/16S_ribosomal_RNA database revealed the highest identities of 99.35% with *P. huaxiensis* strain WCHPr000369 (NR_174258.1). Further phylogenetic analysis of 16S rRNA sequences from all known *Providencia* species indicated that PAZ2 showed closer evolutionary relationship to *P. stuartii* (Fig. 2A). However, given the known limitations of 16S rRNA-based classification for species delineation, we performed additional genomic analyses using ANI and dDDH.

Pairwise genome comparisons between PAZ2 and 16 type strains of known *Providencia* species yielded ANI values ranging from 79.92% to 94.73% and dDDH values from 21.2% to 57.6%, all below the established species threshold (ANI ≥96%, dDDH ≥70%, Table 2). The highest ANI value (94.73%) was observed with *P. zhijiangensis,* suggesting a close but distinct relationship. Notably, our isolate PAZ2 corresponds to the previously identified but unnamed taxon5 from our earlier study (15), with 97.10% ANI and 73.6% dDDH values exceeding the species-defining thresholds. This confirms that PAZ2 represents the formal characterization and naming of this previously recognized but unnamed taxonomic group within *Providencia*.

**TABLE 2:** ANI and dDDH values comparing strain PAZ2 with known *Providencia* species

Species and/or strain	Assembly accession	PAZ2	
		ANI (%)	dDDH (%)
*Providencia stuartii* ATCC 25827	GCA_000154865.1	80.29	21.3
*Providencia manganoxydans* LLDRA6	GCA_016618195.1	80.52	21.9
*Providencia burhodogranariea* DSM 19968	GCA_000314855.2	79.92	21.1
*Providencia sneebia* DSM 19967	GCA_000314895.2	79.94	21.3
*Providencia hangzhouensis* PR-310	GCA_029193595.2	81.25	21.7
*Providencia rettgeri* NCTC11801	GCA_900455085.1	81.63	21.9
*Providencia huaxiensis* WCHPr000369	GCA_002843235.3	81.63	22.2
*Providencia wenzhouensis* R33	GCA_019343475.1	81.22	21.9
*Providencia alcalifaciens* DSM 30120	GCA_000173415.1	87.91	31.3
*Providencia heimbachae* ATCC 35613	GCA_001655055.1	80.84	21.3
*Providencia vermicola* DSM 17385	GCA_020381325.1	81.11	21.2
*Providencia rustigianii* DSM 4541	GCA_000156395.1	83.99	24.4
*Providencia zhijiangensis* D4759	GCA_030315915.2	94.73	57.6
*Providencia xianensis* 23021821	GCA_034661195.1	80.98	21.4
*Providencia xihuensis* SKLX074055	GCA_041953405.1	81.24	21.7
*Providencia zhejiangensis* SKLX146130	GCA_041953975.1	80.37	21.2

To further validate these findings, we constructed phylogenetic trees based on five housekeeping genes and core single-nucleotide polymorphisms (SNPs). Both analyses consistently placed PAZ2 within the genus *Providencia*, clustering with *P. zhijiangensis* (Fig. 2B and C), with branch support values of 100% at major nodes. The substantial branch length separating PAZ2 from *P. zhijiangensis* supports their classification as distinct species, while their clustering in the same phylogenetic branch suggests they share a common evolutionary origin. To further elucidate this relationship, we performed a pan-genome analysis including all *P. lanzhouensis* strains (*n* = 18, including PAZ2) and seven *P. zhijiangensis* strains retrieved from NCBI GenBank. A phylogenetic tree based on core gene alignments clearly resolved the two species into distinct monophyletic branches, with 100% bootstrap support at the node separating them (Fig. S2). Additionally, pairwise ANI values between strains of the two species consistently remained below 94.85%, further reinforcing their delineation as separate species.

### Physiological and metabolic features

**Fig 3:**
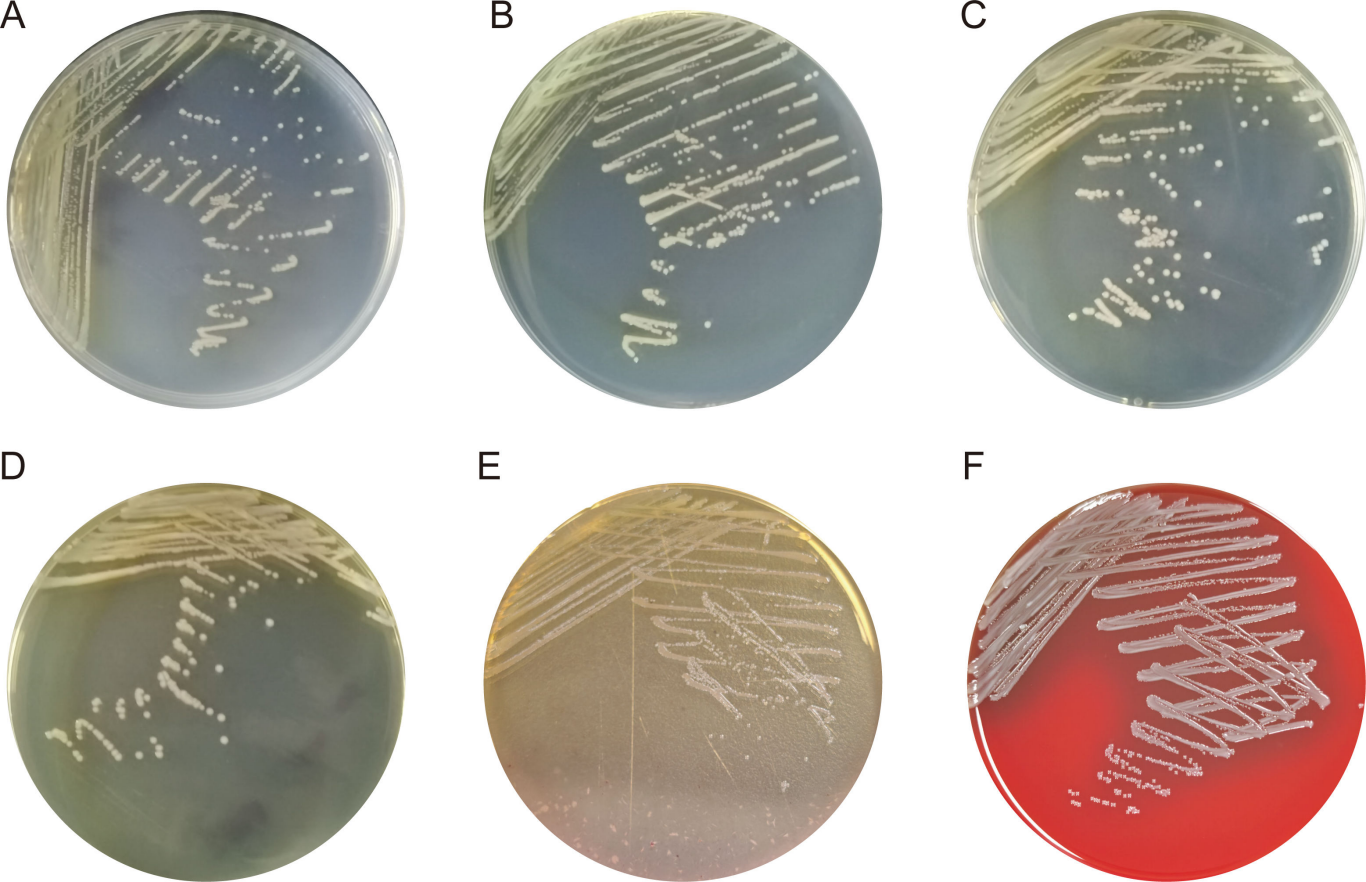
Morphological characteristics of PAZ2. The morphology of the –strain PAZ2 in different culture media is shown in A–D. All bacteria were cultured at 37°C in an aerobic environment. A, Müller-Hinton Agar. B, Luria-Bertani agar. C, Trypticase soy agar. D, Brain Heart Infusion Agar. E, MacConkey (MAC) agar. F, Columbia Blood Agar (CBA).

Table 3 presents a comprehensive comparative analysis of the biochemical profiles of strain PAZ2 against other established *Providencia* species. To determine the optimal incubation conditions for strain PAZ2 growth, we ran a series of growth tests and bioanalyses. The strain PAZ2 was capable of growing on a variety of culture media, including TSA, LBA, BHIA, and MHA at a temperature of 37℃ with ambient air condition. Colonies appeared circular, raised, yellow, opaque, and with a smooth texture (Fig. 3). The temperature range of the strain was determined to be 22–42°C, exhibiting optimal growth at 35 and 37°C. The bacterial cells were capable of surviving within a pH range of 5–9, with an optimal growth observed at pH 6.0–7.0. For salt tolerance, the bacteria were able to thrive within the conditions of 0%–6% (wt/vol) NaCl. Morphologically, the organism comprises Gram-negative, mobile, and facultatively anaerobic rods. Notably, this strain PAZ2 lacked oxidase activity. In comparison to *P. zhijiangensis*, this strain exhibited weaker tolerance to environmental stresses, especially in terms of alkali resistance and salt tolerance.

**TABLE 3:** Biochemical characteristics of strain PAZ2 and type strains of other *Providencia* species^*a*^

Characteristic	PAZ2	1	2	3	4	5	6	7	8	9	10	11	12	13	14	15	16	17	18	19
API 20E tests:																				
β-Galactosidase	−	−	−	−	−	−	−	−	−	−	+	−	−	−	−	−	−	ND	−	−
L-Arginine dihydrolase	−	−	−	−	−	−	−	−	−	−	+	−	−	−	−	−	−	ND	−	−
Lysine decarboxylase	−	−	−	−	−	−	−	−	−	−	−	−	−	−	−	−	−	−	−	−
L-Ornithine decarboxylase	−	−	−	−	−	−	−	−	−	−	+	−	−	−	−	−	−	ND	−	−
Citrate utilization	+	+	−	−	+	+	−	−	−	+	+	−	+	+	+	+	+	+	+	+
H_2_S production	−	−	−	−	−	−	−	−	−	−	−	−	−	−	−	−	−	−	−	−
Urea hydrolysis	−	+	−	−	−	+	+	−	+	−	−	−	−	+	+	+	+	−	+	−
Deaminase	+	+	+	+	+	+	+	+	+	+	+	+	+	+	+	+	+	ND	+	+
Indole production	+	+	+	+	−	+	+	+	+	+	−	+	−	−	−	+	+	−	+	+
Acetoin production	−	−	−	−	−	−	+	−	−	−	+	−	+	−	−	+	−	−	−	−
Gelatinase	−	−	−	−	−	−	−	−	−	−	+	−	−	−	−	−	−	ND	−	−
D-Glucose	+	+	+	+	+	+	+	+	+	+	+	+	+	+	+	+	+	+	+	+
D-Mannitol	−	+	−	+	−	+	−	−	+	−	+	+	+	+	+	+	+	+	+	+
Inositol	−	+	−	+	−	+	+	−	−	+	+	+	−	+	+	+	+	−	+	+
D-Sorbitol	−	−	−	−	−	−	−	−	+	−	+	−	−	−	−	−	−	ND	−	−
L-Rhamnose	−	−	−	−	−	−	+	−	−	−	+	−	−	−	−	+	−	ND	+	−
Sucrose	−	+	−	−	−	−	−	−	−	+	+	−	+	−	−	−	−	ND	−	−
Melibiose	−	−	−	−	−	−	−	−	−	−	+	−	−	−	−	−	−	ND	−	−
Amygdalin	−	−	−	−	−	+	+	−	+	−	−	−	−	+	+	+	+	ND	−	−
L-Arabinose	−	−	−	+	−	−	−	−	−	−	+	+	−	−	−	−	−	ND	−	−
API 50CHE tests:																				
Esculin	−	−	+	+	+	+	+	+	+	−	+	−	−	+	+	ND	ND	ND	+	−
Arbutin	−	−	−	+	+	+	−	−	+	−	+	−	−	+	+	ND	ND	ND	+	−
Cellobiose	−	−	−	−	−	−	−	−	−	−	+	−	−	−	−	ND	ND	ND	−	−
glycerol	−	+	−	−	−	−	+	−	−	+	+	−	+	+	+	ND	ND	−	+	+
2-ketogluconate	−	−	−	−	−	+	−	−	−	−	+	+	−	−	−	ND	ND	ND	−	−
D-Lyxose	−	+	−	−	−	−	−	−	−	+	−	−	−	−	−	ND	ND	ND	−	+
salicin	−	−	−	+	−	+	−	−	+	−	+	−	−	+	+	ND	ND	+	+	−
D-Xylose	−	−	−	−	−	−	−	−	+	−	+	−	−	−	−	ND	ND	ND	−	−

^*a*^ Strains: 1, *P. manganoxydans* LLDRA6; 2, *P. alcalifaciens* DSM 30120; 3, *P. burhodogranariea* DSM 19968; 4, *P. heimbachae* DSM 3591; 5, *P. huaxiensis* KCTC 62577; 6, *P. rettgeri* DSM4542; 7, *P. rustigianii* DSM 4541; 8, *P. sneebia* DSM 19967; 9, *P. stuartii* DSM 4539; 10, *P. thailandensis* KCTC 23281; 11, *P. vermicola* DSM 17385; 12, *P. zhijiangensis* D4759; 13, *P. xianensis* 2302182; 14, *P. huashanensis* CRE-3FA-0001; 15, *P. hangzhouensis* PR-310; 16, *P. entomophila* IO-23; 17, *P. wenzhouensis* R33; 18, *P. xihuensis* SKLX74055; 19, *P. zhejiangensis* SKLX146130. ND, not determine.

### Global distribution of the novel species

To investigate the global distribution of *P. lanzhouensis*, we analyzed available genomic data from the NCBI GenBank database. Our analysis identified 17 strains showing high genomic similarity to *P. lanzhouensis* PAZ2. Among all 18 strains (including PAZ2), the majority were isolated from China (27.8%, 5/18), with additional strains discovered in Lebanon, the United States, Poland, and France. Human-derived isolates accounted for 38.9% (7/18) of all strains, predominantly from China and the United States, while the remaining strains were isolated from diverse environmental sources including flies and food samples across different geographical locations (Table 4).

**TABLE 4:** Distribution of *P. lanzhouensis* sp. nov. and closely related novel strains^*a*^

Assembly	Strain	Location	Collection date	Host/source	Reference
PAZ2	PAZ2	China	2024-03-21	Human	This study
GCA_000517745.1	PAL-3	Unknown	Unknown	Unknown	(1)
GCA_000527275.1	PAL-1	Unknown	Unknown	Unknown	(1)
GCA_003057415.1	PRM-2	Lebanon	2017-09-05	Human	(1)
GCA_004343335.1	JUb102	Unknown	Unknown	Unknown	(1)
GCA_902375285.1	MGYG-HGUT-01465	Unknown	Unknown	Human	(1)
GCA_015739385.1	PRV00016	USA	2018	Human	(1)
GCA_009706335.1	wls1938	Poland	Unknown	Unknown	(2)
GCA_009706495.1	wls1921	Poland	Unknown	Unknown	(2)
GCA_009706505.1	wls1922	Poland	Unknown	Unknown	(2)
GCA_009706585.1	wls1916	Poland	Unknown	Unknown	(2)
GCA_012272845.2	JUb39	France	2019-11-01	Food	(3)
GCA_018257355.1	JGM178	USA	2015	Fly	(4)
GCA_018257405.1	JGM172	USA	2015	Fly	(4)
GCA_027595165.1	PROV188	China	2016	Human	(5)
GCA_028479405.1	PROV212	China	Unknown	Human	(5)
GCA_028479425.1	PROV211	China	Unknown	Human	(5)
GCA_040139585.1	fly-1054	China	2023	Fly	(6)

^*a*^ NCBI search; 2: (37); 3: (38); 4: (39); 5: (40); 6: (41).

**Fig 4:**
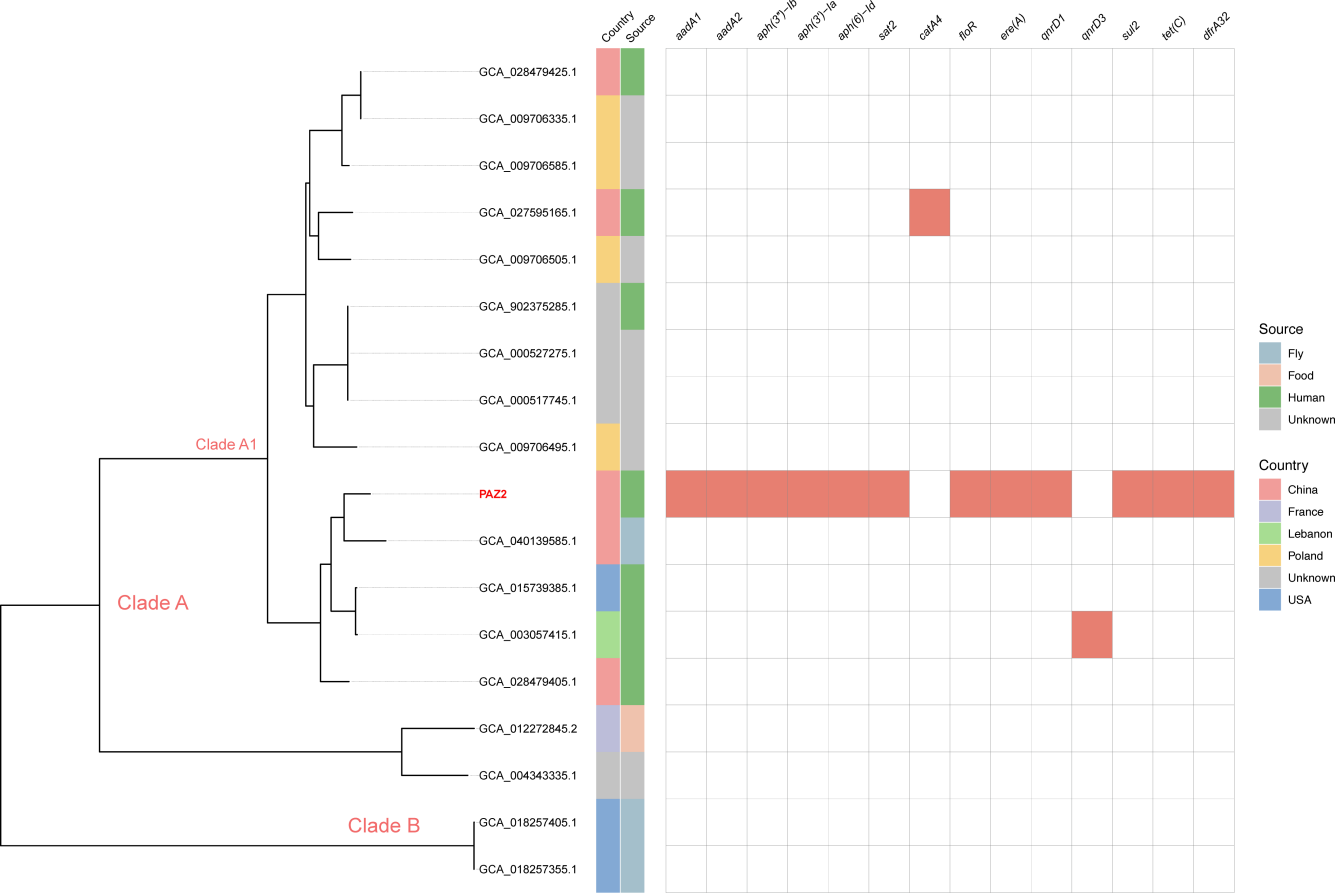
Phylogenetic tree of species *P. lanzhouensis* and distribution of drug-resistance genes. The distribution of resistance genes is shown as a heat map, with blanks indicating the absence of the gene.

Phylogenetic analysis revealed that these strains clustered into two major clades: clade A and clade B. Clade B exclusively comprised strains isolated from flies in the United States, while clade A showed greater geographical and host diversity. Clade A is further subdivided into two distinct sub-clades (A1 and A2), with strain PAZ2 clustering within sub-clade A1 (Fig. 4). Comparative analysis of antimicrobial resistance genes demonstrated that *P. lanzhouensis* PAZ2 possessed a distinctively comprehensive resistance profile compared to other strains in the analysis. PAZ2 harbored multiple resistance determinants, including genes conferring resistance to aminoglycosides (*aadA1*, *aadA2*, *aph(3'')-Ib*, *aph(3')-Ia*, *aph(6)-Id*), *streptomycin* (*sat2*), *sulfonamides* (*sul2*), and tetracyclines (*tet(C*)). In contrast, among the other 17 strains analyzed, only two strains carried a single resistance gene each, while the remaining strains lacked detectable resistance determinants (Fig. 4).

## DISCUSSION

Through mass spectrometry, we initially classified a clinical strain as belonging to the *Providencia* genus, but it was misidentified as a *P. alcalifaciens* species. Whole-genome sequencing later revealed that the strain shares the highest ANI (94.73%) and dDDH (57.6%) values with *P. zhijiangensis*. Although early studies suggested that bacterial species thresholds lie between 95% and 96% ANI (42), more recent analyses—such as those by Ciufo et al. (43) and Rodriguez et al. (44)—support the adoption of a stricter 96% ANI cutoff. These studies demonstrated that intraspecies ANI values among prokaryotes consistently fall within the 96%–100% range, providing evolutionary and statistical justification for this threshold. Likewise, a dDDH value of 70% is widely accepted as the species boundary (45). Given that both values between our strain and *P. zhijiangensis* fall below these thresholds, the strain cannot be assigned to any currently described Providencia species. Based on comprehensive genomic and phenotypic characterization, we, therefore, propose the designation of *Providencia lanzhouensis* sp. nov. This finding adds to the understanding of species boundaries within Providencia, enhances bacterial taxonomy, and contributes to improved accuracy in clinical pathogen identification.

Notably, similar misidentification by MALDI-TOF MS has been observed in the identification of *P. zhijiangensis* and *P. hangzhouensis* (14, 15). These systematic errors highlight the limitations of current mass spectrometry-based identification systems, likely resulting from the rapid bacterial evolution and delayed updates of classification databases. This phenomenon emphasizes the necessity of integrating whole-genome sequencing and biochemical analyses for comprehensive identification in clinical diagnostics, while also underscoring the urgent need for developing more accurate identification systems. A more accurate identification system can be achieved by optimizing the mass spectrometry database for clinically relevant strains and employing machine learning algorithms to enhance spectral recognition capabilities, thereby reducing species misidentification.

Compared to the low support observed in the 16S rRNA phylogenetic tree (Fig. 2A), the MLSA and core SNP-based trees (Fig. 2B and C) exhibited strong bootstrap support (>90%) for nearly all nodes, highlighting their superior suitability for species delineation. In particular, the whole-genome-based SNP tree offers the highest phylogenetic resolution. Notably, one node in the MLSA tree displayed a bootstrap value below 80%, suggesting minor uncertainty in that specific branching. This may stem from the high sequence conservation of the housekeeping genes used in MLSA, which can limit resolution at certain points. Nevertheless, the overall topological congruence between the MLSA and SNP trees—together with ANI and dDDH values and distinct phenotypic traits—strongly supports the designation of *P. lanzhouensis* as a novel species. Future inclusion of additional loci or full-genome phylogenomic approaches may further improve the resolution of MLSA-based trees.

Despite the close phylogenetic relationship between *P. lanzhouensis* and *P. zhijiangensis*, comparative genomic analyses—including pan-genome-based phylogeny and ANI comparisons—consistently delineate the two as distinct species. This genomic divergence is further supported by phenotypic differentiation, as *P. lanzhouensis* displays unique metabolic characteristics not observed in *P. zhijiangensis*. Such congruent separation at both the genomic and phenotypic levels underscore the importance of multi-layered taxonomic frameworks that integrate evolutionary, functional, and ecological perspectives. The observed metabolic differences may reflect niche-specific adaptations or functional specialization, suggesting that divergence between these species is driven not only by sequence-level changes but also by selective pressures shaping their physiological capabilities. These findings warrant further investigation into the ecological niches occupied by each species and how environmental or host-associated factors may have contributed to their evolutionary trajectories.

A striking feature of *P. lanzhouensis* PAZ2 is its extensive antimicrobial resistance profile, which contrasts sharply with the limited or absent resistance determinants in closely related strains. PAZ2 harbors multiple resistance genes, conferring resistance to aminoglycosides, streptomycin, sulfonamides, and tetracyclines, indicative of significant adaptive evolution. The underlying genetic architecture points to horizontal gene transfer (HGT) as a major contributor, particularly for chromosomal resistance genes—all of which are associated with insertion sequences or integrons. These mobile genetic elements likely facilitated the capture and integration of diverse resistance genes, enabling PAZ2 to persist in antimicrobial-rich environments. Integrons, in particular, are well-known for mediating the acquisition of resistance gene cassettes (46), and their presence in PAZ2 reflects a dynamic evolutionary process shaped by sustained antibiotic pressure in clinical or ecological niches. In contrast, the *qnrD1* gene is carried on a small non-conjugative plasmid that lacks flanking mobile elements. However, this plasmid shares high sequence similarity with the previously characterized *qnrD1*-carrying plasmid pDIJ09-518a from *P. rettgeri*. Despite its limited intrinsic mobility, the potential for dissemination via helper plasmids or homologous recombination events cannot be ruled out. Together, these findings underscore the combined influence of chromosomal and extrachromosomal mechanisms in shaping the multidrug resistance landscape of *P. lanzhouensis* and emphasize the importance of monitoring mobile genetic elements in clinical pathogens.

Our study has several limitations. First, the relatively small sample size (18 strains) and geographic bias—particularly the predominance of strains from China—likely reflect regional sampling patterns in currently available genomic data rather than the true global distribution of *P. lanzhouensis*. While this data set allowed for initial insights into the species’ genetic characteristics and clinical relevance, it limits the generalizability of findings regarding population structure and global diversity. Second, environmental sources such as water or soil were not included, despite their potential role as reservoirs in the dissemination of antimicrobial resistance genes. Broader sampling across ecological niches and geographic regions will be essential to better understand the species’ distribution and evolution. Finally, this study did not perform detailed genotype–phenotype correlation analyses, such as linking metabolic capabilities to annotated genes, as the primary focus was on taxonomic and phenotypic characterization. We recognize the importance of this approach and plan to address it in future functional studies.

In summary, the identification of *P. lanzhouensis* sp. nov. advances the taxonomy of the *Providencia* genus while exposing deficiencies in current identification systems, particularly the limitations of MALDI-TOF MS for novel species. The pronounced antimicrobial resistance of PAZ2 poses new challenges for clinical management, emphasizing the need for continued research into its resistance mechanisms, pathogenicity, and prevalence. To better understand *P. lanzhouensis*, future research should prioritize virulence studies to elucidate its pathogenic potential, including the identification of virulence factors and infection mechanisms in clinical settings. Additionally, expanded genomic surveys, such as comparative genomics across multiple *P. lanzhouensis* strains and related *Providencia* species, are essential to uncover evolutionary dynamics, resistance gene dissemination, and ecological adaptations. Further investigation into horizontal gene transfer mechanisms and environmental drivers of resistance will also be critical to inform strategies for mitigating its clinical impact. By integrating genomic, phenotypic, and ecological perspectives, these studies can further illuminate the evolutionary trajectory and clinical relevance of this emerging pathogen.

### Description of *Providencia lanzhouensis* sp. nov.

*P. lanzhouensis* (lan.zhou.en’sis. N.L. fem. adj. *lanzhouensis*, referring to Lanzhou City, Gansu Province, China, where the type strain was first isolated).

The cells are Gram-negative, motile, facultatively anaerobic rods, catalase-positive and oxidase-negative. The strain demonstrates robust growth on various culture media, including TSA, CBA, MAC, LB, BHI, and MH agar, with optimal growth observed at 37°C under aerobic conditions. C–olonies are circular, raised, yellow, opaque, and smooth in texture. Growth occurs across a pH range of 5–9, with an optimal range between pH 6 and 7, and in NaCl concentrations ranging from 0 to 6% (wt/vol). Biochemical tests reveal positive results for deaminase activity and the fermentation of D-glucose, as well as positive results for citrate utilization.

The type strain, PAZ2^T^ = CCTCC AB 2024284^T^ = KCTC 8881^T^, was isolated from a urine specimen from Gansu Province, China. The DNA G  +  C content of the strain is 42.8%.

## Data Availability

The complete genome sequence of PAZ2 from this study has been deposited in the GenBank database under BioProject accession PRJNA1180771.

## SUPPLEMENTAL MATERIAL

Supplemental figures spectrum.00549-25-s0001.pdf Figures S1 and S2.

ASM does not own the copyrights to Supplemental Material that may be linked to, or accessed through, an article. The authors have granted ASM a non-exclusive, world-wide license to publish the Supplemental Material files. Please contact the corresponding author directly for reuse.

## References

[1] Adeolu M, Alnajar S, Naushad S, S Gupta R. 2016. Genome-based phylogeny and taxonomy of the “Enterobacteriales”: proposal for Enterobacterales ord. nov. divided into the families Enterobacteriaceae, Erwiniaceae fam. nov., Pectobacteriaceae fam. nov., Yersiniaceae fam. nov., Hafniaceae fam. nov., Morganellaceae fam. nov., and Budviciaceae fam. nov. Int J Syst Evol Microbiol 66:5575–5599. doi:10.1099/ijsem.0.001485 27620848

[2] Wie SH. 2015. Clinical significance of Providencia bacteremia or bacteriuria. Korean J Intern Med 30:167–169. doi:10.3904/kjim.2015.30.2.167 25750557 PMC4351322

[3] O’Hara CM, Brenner FW, Miller JM. 2000. Classification, identification, and clinical significance of Proteus, Providencia, and Morganella. Clin Microbiol Rev 13:534–546. doi:10.1128/CMR.13.4.534 11023955 PMC88947

[4] Brenner DJ, Farmer JJ, Fanning GR, Steigerwalt AG, Klykken P, Wathen HG, et al.. 1978. Deoxyribonucleic acid relatedness of Proteus and Providencia species. Int J Syst Evol Microbiol 28:269–282.

[5] Warren JW. 1986. Providencia stuartii: a common cause of antibiotic-resistant bacteriuria in patients with long-term indwelling catheters. Rev Infect Dis 8:61–67. doi:10.1093/clinids/8.1.61 3081988

[6] Hickman-Brenner FW, Farmer JJ 3rd, Steigerwalt AG, Brenner DJ. 1983. Providencia rustigianii: a new species in the family Enterobacteriaceae formerly known as Providencia alcalifaciens biogroup 3. J Clin Microbiol 17:1057–1060. doi:10.1128/jcm.17.6.1057-1060.1983 6874899 PMC272801

[7] Müller HE, O’hara CM, Fanning GR, Hickman-Brenner FW, Swenson JM, Brenner DJ. 1986. Providencia heimbachae, a new species of Enterobacteriaceae isolated from animals. Int J Syst Evol Microbiol 36:252–256.

[8] Somvanshi VS, Lang E, Sträubler B, Spröer C, Schumann P, Ganguly S, Saxena AK, Stackebrandt E. 2006. Providencia vermicola sp. nov., isolated from infective juveniles of the entomopathogenic nematode Steinernema thermophilum. Int J Syst Evol Microbiol 56:629–633. doi:10.1099/ijs.0.63973-0 16514040

[9] Juneja P, Lazzaro BP. 2009. Providencia sneebia sp. nov. and Providencia burhodogranariea sp. nov., isolated from wild Drosophila melanogaster. Int J Syst Evol Microbiol 59:1108–1111. doi:10.1099/ijs.0.000117-0 19406801

[10] Khunthongpan S, Sumpavapol P, Tanasupawat S, Benjakul S, H-Kittikun A. 2013. Providencia thailandensis sp. nov., isolated from seafood processing wastewater. J Gen Appl Microbiol 59:185–190. doi:10.2323/jgam.59.185 23863288

[11] Hu Y, Feng Y, Zhang X, Zong Z. 2019. Providencia huaxiensis sp. nov., recovered from a human rectal swab. Int J Syst Evol Microbiol 69:2638–2643. doi:10.1099/ijsem.0.003502 31162027

[12] Ksentini I, Gharsallah H, Sahnoun M, Schuster C, Hamli Amri S, Gargouri R, Triki MA, Ksantini M, Leclerque A. 2019. Providencia entomophila sp. nov., a new bacterial species associated with major olive pests in Tunisia. PLoS One 14:e0223943. doi:10.1371/journal.pone.0223943 31639141 PMC6805009

[13] Li Z, Liao F, Ding Z, Chen S, Li D. 2022. Providencia manganoxydans sp. nov., a Mn(II)-oxidizing bacterium isolated from heavy metal contaminated soils in Hunan Province, China. Int J Syst Evol Microbiol 72. doi:10.1099/ijsem.0.005474 35930465

[14] Dong X, Yu Y, Liu J, Cao D, Xiang Y, Bi K, Yuan X, Li S, Wu T, Zhang Y. 2023. Whole-genome sequencing provides insights into a novel species: Providencia hangzhouensis associated with urinary tract infections. Microbiol Spectr 11:e0122723. doi:10.1128/spectrum.01227-23 37732781 PMC10581081

[15] Dong X, Jia H, Yu Y, Xiang Y, Zhang Y. 2024. Genomic revisitation and reclassification of the genus Providencia. mSphere 9:e0073123. doi:10.1128/msphere.00731-23 38412041 PMC10964429

[16] Dong Xu, Xiang Y, Yang P, Wang S, Yan W, Yuan Y, Zhou S, Zhou K, Liu J, Zhang Y. 2024. Novel Providencia xianensis sp. nov.: a multidrug-resistant species identified in clinical infections. Eur J Clin Microbiol Infect Dis 43:1461–1467. doi:10.1007/s10096-024-04821-y 38714595 PMC11271419

[17] Zhou K, Liang J, Dong X, Zhang P, Feng C, Shi W, Gao M, Li Q, Zhang X, Lu J, Lin X, Li K, Zhang H, Zhu M, Bao Q. 2021. Identification and characterization of a novel chromosomal aminoglycoside 2′-N-acetyltransferase, AAC(2′)-If, from an isolate of a novel Providencia species, Providencia wenzhouensis R33. Front Microbiol 12:711037. doi:10.3389/fmicb.2021.711037 34867838 PMC8640171

[18] Yang W, Chen J, Yang F, Ji P, Shen S, Yin D, Hu F. 2024. Identification of a novel Providencia species showing multi-drug-resistant in three patients with hospital-acquired infection. Int J Antimicrob Agents 64:107211. doi:10.1016/j.ijantimicag.2024.107211 38795927

[19] Dong X, Xiang Y, Shen P, Xiao Y, Zhang Y. 2025. Clinical emergence of Providencia zhejiangensis sp. nov. and Providencia xihuensis sp. nov.: genomic insights into antimicrobial resistance and geographical distribution. Int J Antimicrob Agents 65:107484. doi:10.1016/j.ijantimicag.2025.107484 40023453

[20] Edwards LD, Cross A, Levin S, Landau W. 1974. Outbreak of a nosocomial infection with a strain of Proteus rettgeri resistant to many antimicrobials. Am J Clin Pathol 61:41–46. doi:10.1093/ajcp/61.1.41 4358334

[21] Mulet M, Lalucat J, García-Valdés E. 2010. DNA sequence-based analysis of the Pseudomonas species. Environ Microbiol 12:1513–1530. doi:10.1111/j.1462-2920.2010.02181.x 20192968

[22] Humphries R, Bobenchik AM, Hindler JA, Schuetz AN. 2021. Overview of changes to the clinical and laboratory standards institute Performance standards for antimicrobial susceptibility testing, M100, 31st Edition. J Clin Microbiol 59:e0021321. doi:10.1128/JCM.00213-21 34550809 PMC8601225

[23] Wick RR, Judd LM, Gorrie CL, Holt KE. 2017. Unicycler: resolving bacterial genome assemblies from short and long sequencing reads. PLoS Comput Biol 13:e1005595. doi:10.1371/journal.pcbi.1005595 28594827 PMC5481147

[24] Jain C, Rodriguez-R LM, Phillippy AM, Konstantinidis KT, Aluru S. 2018. High throughput ANI analysis of 90K prokaryotic genomes reveals clear species boundaries. Nat Commun 9:5114. doi:10.1038/s41467-018-07641-9 30504855 PMC6269478

[25] Meier-Kolthoff JP, Carbasse JS, Peinado-Olarte RL, Göker M. 2022. TYGS and LPSN: a database tandem for fast and reliable genome-based classification and nomenclature of prokaryotes. Nucleic Acids Res 50:D801–D807. doi:10.1093/nar/gkab902 34634793 PMC8728197

[26] Meier-Kolthoff JP, Auch AF, Klenk H-P, Göker M. 2013. Genome sequence-based species delimitation with confidence intervals and improved distance functions. BMC Bioinformatics 14:60. doi:10.1186/1471-2105-14-60 23432962 PMC3665452

[27] Feldgarden M, Brover V, Gonzalez-Escalona N, Frye JG, Haendiges J, Haft DH, Hoffmann M, Pettengill JB, Prasad AB, Tillman GE, Tyson GH, Klimke W. 2021. AMRFinderPlus and the reference gene catalog facilitate examination of the genomic links among antimicrobial resistance, stress response, and virulence. Sci Rep 11:12728. doi:10.1038/s41598-021-91456-0 34135355 PMC8208984

[28] Schwengers O, Jelonek L, Dieckmann MA, Beyvers S, Blom J, Goesmann A. 2021. Bakta: rapid and standardized annotation of bacterial genomes via alignment-free sequence identification. Microb Genom 7:000685. doi:10.1099/mgen.0.000685 34739369 PMC8743544

[29] Weisburg WG, Barns SM, Pelletier DA, Lane DJ. 1991. 16S ribosomal DNA amplification for phylogenetic study. J Bacteriol 173:697–703. doi:10.1128/jb.173.2.697-703.1991 1987160 PMC207061

[30] Yoon S-H, Ha S-M, Kwon S, Lim J, Kim Y, Seo H, Chun J. 2017. Introducing EzBioCloud: a taxonomically united database of 16S rRNA gene sequences and whole-genome assemblies. Int J Syst Evol Microbiol 67:1613–1617. doi:10.1099/ijsem.0.001755 28005526 PMC5563544

[31] Katoh K, Misawa K, Kuma K, Miyata T. 2002. MAFFT: a novel method for rapid multiple sequence alignment based on fast Fourier transform. Nucleic Acids Res 30:3059–3066. doi:10.1093/nar/gkf436 12136088 PMC135756

[32] Capella-Gutiérrez S, Silla-Martínez JM, Gabaldón T. 2009. trimAl: a tool for automated alignment trimming in large-scale phylogenetic analyses. Bioinformatics 25:1972–1973. doi:10.1093/bioinformatics/btp348 19505945 PMC2712344

[33] Price MN, Dehal PS, Arkin AP. 2010. FastTree 2--approximately maximum-likelihood trees for large alignments. PLoS One 5:e9490. doi:10.1371/journal.pone.0009490 20224823 PMC2835736

[34] Yu G, Huachen Z, Yi G, Tommy Tsan-Yuk L, David KS. 2017. Ggtree: an R package for visualization and annotation of phylogenetic trees with their covariates and other associated data. Methods Ecol Evol 8

[35] Tonkin-Hill G, MacAlasdair N, Ruis C, Weimann A, Horesh G, Lees JA, Gladstone RA, Lo S, Beaudoin C, Floto RA, Frost SDW, Corander J, Bentley SD, Parkhill J. 2020. Producing polished prokaryotic pangenomes with the Panaroo pipeline. Genome Biol 21:180. doi:10.1186/s13059-020-02090-4 32698896 PMC7376924

[36] Guillard T, Cambau E, Neuwirth C, Nenninger T, Mbadi A, Brasme L, Vernet-Garnier V, Bajolet O, de Champs C. 2012. Description of a 2,683-base-pair plasmid containing qnrD in two Providencia rettgeri isolates. Antimicrob Agents Chemother 56:565–568. doi:10.1128/AAC.00081-11 21986831 PMC3256040

[37] Yuan C, Wei Y, Zhang S, Cheng J, Cheng X, Qian C, Wang Y, Zhang Y, Yin Z, Chen H. 2020. Comparative genomic analysis reveals genetic mechanisms of the variety of pathogenicity, antibiotic resistance, and environmental adaptation of Providencia Genus. Front Microbiol 11:572642. doi:10.3389/fmicb.2020.572642 33193173 PMC7652902

[38] Bubrig LT, Sutton JM, Fierst JL. 2020. Caenorhabditis elegans dauers vary recovery in response to bacteria from natural habitat. Ecol Evol 10:9886–9895. doi:10.1002/ece3.6646 33005351 PMC7520223

[39] McMullen JG, Bueno E, Blow F, Douglas AE. 2021. Genome-inferred correspondence between phylogeny and metabolic traits in the wild Drosophila gut microbiome. Genome Biol Evol 13:evab127. doi:10.1093/gbe/evab127 34081101 PMC8358223

[40] Wang P, Li C, Yin Z, Jiang X, Li X, Mu X, et al.. 2023. Genomic epidemiology and heterogeneity of Providencia and their bla(NDM-1)-carrying plasmids. Emerg Microbes Infect 12:2275596. doi:10.1080/22221751.2023.2275596 37874004 PMC10796120

[41] Zhou H, Wang H, Chen K, Xie M, Yan Z, Zhang Y, Wu Y, Liu D, Wang J, Dong N, Cai C, Wu Y, Walsh TR, Chen S, Wang Y, Zhang R. 2024. Epidemiological and genomic analysis revealed the significant role of flies in dissemination of carbapenem-resistant Enterobacteriaceae (CRE) in China. J Hazard Mater 480:136374. doi:10.1016/j.jhazmat.2024.136374 39509877

[42] Richter M, Rosselló-Móra R. 2009. Shifting the genomic gold standard for the prokaryotic species definition. Proc Natl Acad Sci U S A 106:19126–19131. doi:10.1073/pnas.0906412106 19855009 PMC2776425

[43] Ciufo S, Kannan S, Sharma S, Badretdin A, Clark K, Turner S, Brover S, Schoch CL, Kimchi A, DiCuccio M. 2018. Using average nucleotide identity to improve taxonomic assignments in prokaryotic genomes at the NCBI. Int J Syst Evol Microbiol 68:2386–2392. doi:10.1099/ijsem.0.002809 29792589 PMC6978984

[44] Rodriguez-R LM, Conrad RE, Viver T, Feistel DJ, Lindner BG, Venter SN, Orellana LH, Amann R, Rossello-Mora R, Konstantinidis KT. 2024. An ANI gap within bacterial species that advances the definitions of intra-species units. MBio 15:e0269623. doi:10.1128/mbio.02696-23 38085031 PMC10790751

[45] Goris J, Konstantinidis KT, Klappenbach JA, Coenye T, Vandamme P, Tiedje JM. 2007. DNA-DNA hybridization values and their relationship to whole-genome sequence similarities. Int J Syst Evol Microbiol 57:81–91. doi:10.1099/ijs.0.64483-0 17220447

[46] Kaushik M, Kumar S, Kapoor RK, Virdi JS, Gulati P. 2018. Integrons in Enterobacteriaceae: diversity, distribution and epidemiology. Int J Antimicrob Agents 51:167–176. doi:10.1016/j.ijantimicag.2017.10.004 29038087

