# Supplementary material for: Taxonomic and phenotypic characterization of a novel Providencia species: Providencia lanzhouensis sp. nov
Source: Microbiol Spectr. 2025 Jul 9;13(8):e00549-25. doi: 10.1128/spectrum.00549-25 (PMC12323597; doi:10.1128/spectrum.00549-25)
Supplement: Supplemental figures — Figures S1 and S2. [file spectrum.00549-25-s0001.pdf]

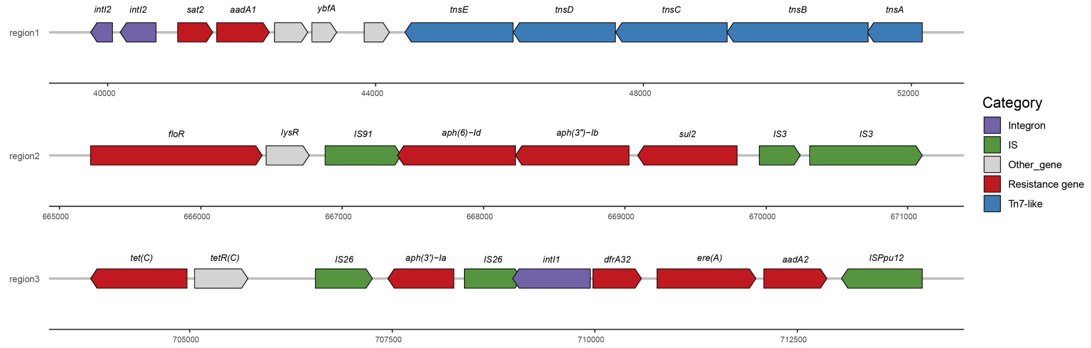

Figure S1. Schematic diagram of the genetic organization of region with resistance genes in PAZ2. Genes are color-coded based on their function.

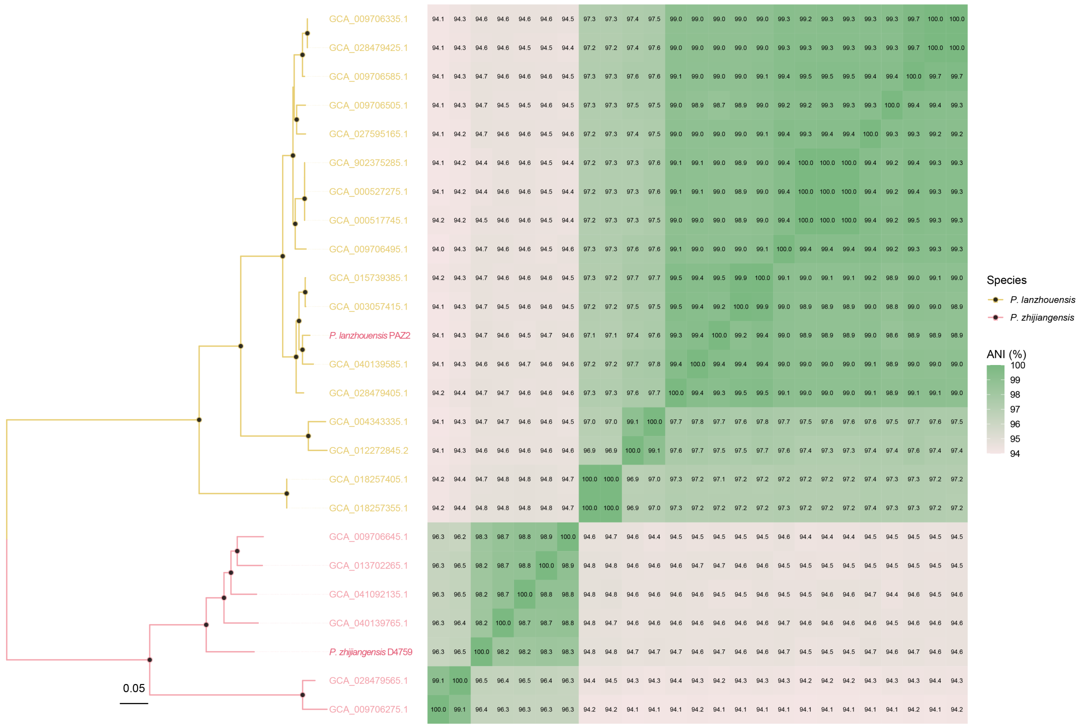

Figure S2. Phylogenetic tree of genomes closely related to PAZ2 and *P. zhijiangensis* D4759. ANI comparisons for each genome are shown on the right side of the tree. Bootstrap values above 90% are indicated by black circles.
